# Recognition and Analysis of Sports on Mental Health Based on Deep Learning

**DOI:** 10.3389/fpsyg.2022.897642

**Published:** 2022-06-15

**Authors:** LingSong Li, HaiXia Li

**Affiliations:** ^1^School of Physical Education, Harbin University, Harbin, China; ^2^Harbin Institute of Physical Education, Harbin, China

**Keywords:** deep learning, athletic sports, mental health, state identification, characteristics of human health mutual information

## Abstract

This paper presents the purpose of sport recognition of mental health for users and analyzes and studies the recognition of mental health by sports based on deep learning. The recognition model of sport mental health state composed of data layer, logic layer and display layer is built. After fusing human health data with deep learning algorithm, the feature of human health mutual information is extracted, the feature into the recognition model of mental health state is inputted, and the recognition results of sport mental health mode after forward and reverse operation are outputted. The recognition data of sports on mental health status are obtained, which correspond to the link flowing through during multi-level transmission, calibrate the multi-level transmission point, and fuse and process the recognition information of sports on mental health status. The experimental results show that the loss value of the research method when analyzing the effect of sports on mental health enhancement is the smallest, the output result is reliable, can effectively improve the body mass index (BMI) of the human body, has the most controllable amount of data, and has good performance.

## Introduction

Human health includes not only the physiological level, but also the psychological level. Physiological and mental health interact and complement each other (Pardos et al., 2021). With the continuous development of social civilization, people’s understanding and research on the content and importance of mental health are also deepening (Bessonov and Nametkin, 2020; Kumar et al., 2021). Mental health has its external performance and internal characteristics. Good social adaptation is its external performance, and good psychological quality is its internal characteristics, especially the level of mental health (Simdiankin and Zhavoronkov, 2020). Therefore, recognition of mental health status is an inevitable outcome of the development of the times, social progress, and the continuous improvement of people’s understanding of their own health in the context of deepening social practice (Patel et al., 2020; Hx et al., 2021). Human quality includes scientific and cultural qualities, moral and ideological qualities, labor ability quality, psychological quality, and physical quality, etc. These qualities can interact (Vegara-Ferri et al., 2020). Among them, psychological quality is the carrier of other qualities and the basis and premise of improving people’s overall quality. Therefore, the cultivation of psychological quality should be the foundation project of quality education (Polancic et al., 2020). As a high-level goal pursued by the cultivation of psychological quality, good mental health constitutes the high-level realm pursued by the development of psychological quality (Lee et al., 2020; Bulbul and Ali, 2021). Quality education should cultivate and shape the various qualities of college students, so that people’s various qualities can be improved accordingly. As a foundation project of psychological quality, its fundamental purpose is to cultivate people with good mental health. Therefore, the development of mental health reflects the essential requirements of quality education.

Schweizer et al. (2020) takes achievement measurement as an example and puts forward reliable measurement methods in sport psychology. This research solves a neglected aspect of the spirit of the times, that is, to improve the method standard of sport psychology. This paper discusses and emphasizes the importance of reliable measurement from different angles and empirically evaluates the reliability of three common performance result measurements, so as to provide guidance for researchers on how to improve the reliability of performance result measurement. In the basketball game, three indexes are studied; in the whole sample, the highest reliability of darts is parity reliability (0.888), followed by golf putter (0.714 away from the hole, 0.614 successful putter), and free throw (0.504 for non-basketball players, 0.62 for basketball players, and 0.826 for the whole sample), so as to improve the reliability of performance measurement of sport psychology. Madsen et al. (2020) puts forward that can the psychological characteristics, football experience, and player status of Danish elite female football players predict the state of anxiety before important matches? Elite football will make players feel nervous, and personality characteristics and experience will affect the handling of important pre-match stress. Studying the psychological characteristics of female football players can provide information on how to deal with psychological stress and generate knowledge on how to support players to improve performance. In this study, 128 elite female football players from 8 top teams have been taken as the samples to study the psychological characteristics of elite female football players and whether football experience or player status can predict the state of anxiety before important matches. The results summarized the negative predictions of high age and national team experience on most trait anxiety subscales. Consistent with previous studies, no psychological differences were found among goalkeepers, defenders, midfielders, and strikers, whereas the trait anxiety of starting players was significantly reduced. When measured before important games, it was found that physical state of anxiety was negatively correlated with the experience of the national team and positively correlated with anxiety, trait anxiety, and fear of failure. Cognitive state of anxiety was negatively correlated with the hope of success and positively correlated with physical anxiety and worry trait anxiety. Self-confidence is positively correlated with the experience of the youth national team and negatively correlated with anxiety. It can be concluded that psychological characteristics and national team experience are very important for the best state of anxiety of excellent women’s football before important competitions. At the same time, the enlightenment to practice and future research is discussed.

Although the above research has made some progress, the research on artificial intelligence is not enough. Therefore, this paper puts forward the recognition and analysis of sports on mental health based on deep learning. Deep learning has made many achievements in search technology, data mining, machine learning, machine translation, natural language processing, multimedia learning, voice, recommendation and personalization technology, and other related fields. The recognition model of sports on mental health status is built, using the deep learning algorithm to fuse the human health data, extract the characteristics of human health mutual information, obtain the recognition data of sports on mental health status, correspond to the link flowing through during multi-level transmission, calibrate the multi-level transmission point, and fuse the recognition information of sports on mental health status. Deep learning enables machines to imitate human activities such as audiovisual and thinking, solves many complex problems of sport recognition of mental health, and makes great progress in artificial intelligence-related technologies. When analyzing the effect of sports on mental health enhancement, the loss value is the smallest, the output result is reliable, can effectively improve the BMI of a human body, has the most controllable data, and has good performance.

## Recognition and Analysis of Sports on Mental Health

### Recognition Model Structure of Sports on Mental Health

The concept of the mental health state is defined as certain internal and relatively stable psychological qualities formed by individuals under the joint action of genetics and environment. These psychological qualities affect or determine the psychological, physiological, and social functions of individuals and then affect the mental health state of individuals (Alotaibi and Alotaibi, 2020; Drager et al., 2020). Combining with the definition of mental health state, the basic characteristics of mental health state are summarized as endogenous and stability. (1) Endogenous: mental health state exists inside individuals and is a psychological quality closely related to the mental health level (Ren et al., 2020). Although mental health state is not an independent psychological phenomenon, its influence objectively exists in the psychological aspect of teenagers. In terms of its difference, it is not the existence or non-existence of difference, but the difference between high and low levels. (2) Stability: the attribute of mental health state is psychological quality, but it is a psychological quality closely related to the development of mental health (Jankovi, 2020). Psychological quality itself has the characteristics of congenital heredity and acquired environmental influence, and it interacts with mental health. Once the mental health state is formed, it must be the psychological precipitation obtained after a long time of development, with internalization–externalization–internalization conversion process. Therefore, the stability of mental health state has the characteristics of cross-time and cross-situation.

**FIGURE 1:**
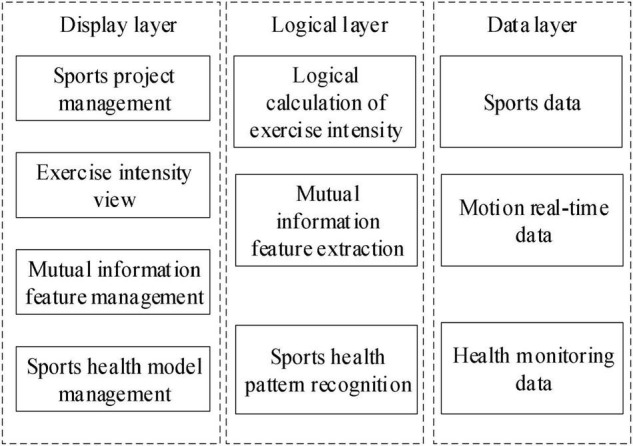
Structure of recognition model of sports on mental health based on deep learning.

Based on the analysis of the factors and evaluation methods of psychological evaluation, this paper discusses the feasibility of establishing a mental health recognition model based on deep learning. The main goal is to study the recognition model based on the psychological evaluation knowledge and the theoretical knowledge of deep learning algorithm and establish a scientific evaluation system. Through the establishment of the mental health status recognition model based on deep learning algorithm, it provides a basis for further establishing a scientific and reasonable qualitative method of mental status, so as to help people realizing their mental status as soon as possible and prevent the occurrence of mental diseases, which makes that the in-depth learning knowledge and recognition model have a certain theoretical level and application value. According to the basic characteristics of mental health state, the recognition model of sports on mental health state based on deep learning is designed, and the structure is shown in Figure 1.

As can be seen from Figure 1, the deep learning-based sport recognition model for mental health status is composed of a data layer, a logic layer, and a display layer. The data layer is used to obtain people’s sport data, real-time sport data, and health identification data and transmit the above data into the logic layer (Ahad et al., 2021). After using the logic layer to perform mutual information feature extraction, exercise health pattern recognition, and exercise intensity logic calculation on people’s exercise data and health data, the relevant calculation results are inputted into the display layer to provide users with exercise project management, exercise intensity viewing, and exercise health model and other information, so that users can fully understand the effect of current exercise on their mental health.

### Feature Extraction of Mental Health Mutual Information

People will generate a large amount of health data during exercise, such as the current pulse rate, blood circulation, body water, and fat changes. The data sources are stored in different formats, forming a multi-source and heterogeneous complex data source (Elumalai and Ramakrishnan, 2020; Kumar and Sukavanam, 2020). To extract useful mental health data from complex data sources, deep learning algorithms are used to fuse multi-source and heterogeneous mental health data and extract mental health mutual information features. The detailed process is as follows:

The scalar time series of mental health data is set as *P* and the dynamic difference feature classification method was used to decompose the scalar time series in window time domain. Its expression formula is as follows:


(1) $$P=\left(A_{a}+B_{b}\right)\times D_{e}\times H_{j}$$

In formula (1), *A*_*a*_ and *B*_*b*_ represent the matching parameters and frequency domain decomposition spectrum, respectively, *D*_*e*_ represents the transient sampling value at the sampling time, and *H*_*j*_ represents the joint distribution state of mutual information of mental health data; fuzzy control is applied to the time-domain sequence in the windowed area of formula (1) to mine the feature vector of mental health data. The state function expression formula of the feature vector is as follows:


(2) $$Q=W_{w}\times \left(R_{1}+R_{2}\right)\times P$$

In formula (2), *W*_*w*_ represents the distribution state vector of mental health data, *R*_*1*_ represents the weight input vector, and *R*_*2*_ represents the distribution distance of feature vector. the deep learning algorithm is used to cluster the time series mean of the results of formula (2) to obtain the linear average time series of mental health data, and its expression is as follows:


(3) $${\bar{O}}_{n}=\frac{\sum_{n=1}^{N}Q}{N}$$

**FIGURE 2:**
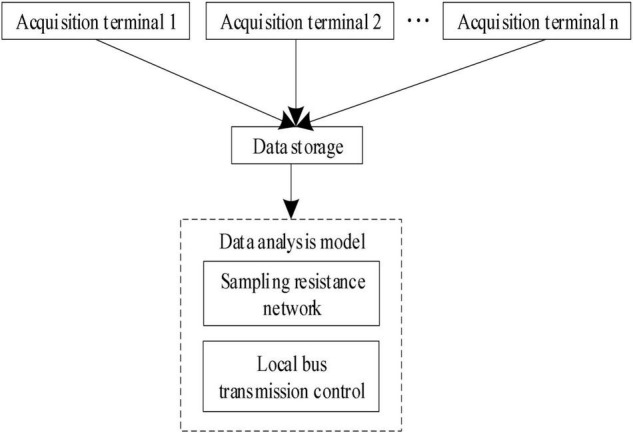
Data acquisition module of sport load state monitoring.

In formula (3), *N* represents the amount of mental health data. After the mutual information features of mental health data are obtained according to the above formula, the deep learning algorithm is used to identify the mutual information features, and the data acquisition module of sport load state monitoring is constructed, as shown in Figure 2.

In the data acquisition module of sport load state monitoring as shown in Figure 2, the local bus transmission control technology is adopted to realize the information fusion of characteristic parameters of sport load state (Dua et al., 2021; Zhang et al., 2021), and the remote transmission control model of characteristic parameters of sport load state is constructed. The TMS320C50 DSP chip is used as the core processing chip of the monitoring system for the state characteristics of sport load to realize the integrated information processing of the state characteristics of sport load and obtain the information characteristics of sport mental health.

### Sport Mental Health Pattern Recognition

Mental health models are divided into three models: Healthy, sub-healthy, and unhealthy (Corral et al., 2021; Javier et al., 2021; Rajbhandari et al., 2021). To clearly show the effect of sports on enhancing mental health after people’s exercise, the three mental health models are divided into three levels: I, II, and III. The smaller the level, the worse the health status in their health model (Cranswick et al., 2020). The mutual information characteristics of the obtained mental health data are inputted into the recognition model of mental health state of sports to identify the mental health mode. The essence of the recognition of mental health mode is the classification and processing of signal sequence. Since sports is a continuous process, its operation can be divided into several cycles (Christou and O’Driscoll, 2020). According to the time relationship of the mutual information characteristics of mental health data, the mental health model is identified to which it belongs to. The detailed process is as follows:

The internal state in the recognition model of mental health state by sports is set and used to record the historical information before the deadline of a certain time and calculate the values of input gate and output gate of long and short memory neural network. The expression formula is as follows:


(4) $$\left\{ \begin{array}{c} S_{r}=\alpha \times U_{r}\times M_{1}\times \lambda \\ S_{c}=\alpha \times U_{c}\times N_{1}\times \lambda \end{array}\right.$$

In formula (4), *S*_*r*_ and *S*_*c*_ represent the mental health characteristic value output by the input gate and output gate, respectively, α represents the activation function, *M*_*1*_ and *N*_*1*_ represent the non-linear activation function and state weight matrix, and λ represents the offset value.

According to the above calculation method, the deep learning algorithm is used to identify the mental health modes of sports, and the steps are as follows:

Step 1: Use formula (4) to calculate the internal state output value set in the recognition model of sports on mental health state.

Step 2: After reverse calculation with formula (5), obtain the error term of mental health status recognition. The formula is as follows:


(5) $$L_{o}=\left(S_{r}+S_{c}\right)\times K_{e\,f}\times B_{z\,x}$$

In formula (5), *K*_*ef*_ represents the output value of hidden layer, *B*_*zx*_ represents the total number of mutual information features of mental health, and *L*_*o*_ represents the classification loss when identifying mental health state patterns.

Step 3: Analyze the feature weight gradient of mental health mutual information according to the result of formula (5).

**FIGURE 3:**
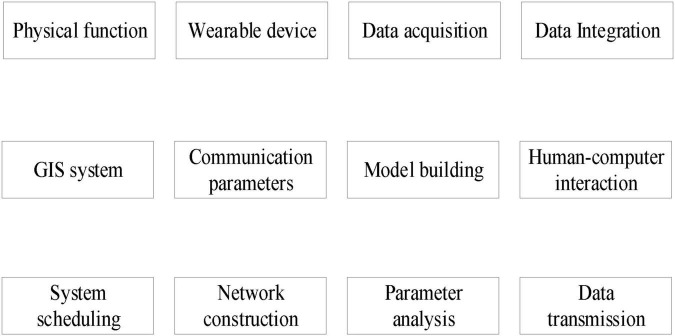
Structure chart of sport mental health pattern recognition.

Step 4: After using the deep learning algorithm to gradient the feature weight of mental health mutual information, re-input the optimization results into the hidden layer of the sport mental health state recognition model (Hsiao and Wang, 2020; Csulak et al., 2021), output the mental health pattern recognition results after reverse calculation, and obtain the mental health pattern recognition structure of sports, as shown in Figure 3.

It can be seen from Figure 3 that the psychological status of each independent mental health assessment object is a multi-dimensional information system. Its basic characteristics are multivariable, multi-level, and strong coupling. There are complex non-linear interactions among various factors in the system. More accurate identification results can be obtained using system analysis.

## Realize the Recognition and Analysis of Sports on Mental Health Based on Deep Learning

The recognition data of sports on mental health status are obtained, which correspond to the link flowing through during multi-level transmission, calibrate the multi-level transmission point (Roberts et al., 2020), and calculate the path loss generated during channel transmission. The numerical relationship can be expressed as follows:


(6) $$V=\frac{V_{a}-V_{b}}{Z_{a\,b}}\times L_{o}$$

In formula (6), *V*_*a*_ represents the total transmission power of the detection data channel, *V*_*b*_ represents the power of the channel receiving identification data, and *Z*_*ab*_ represents the channel gain. Combined with the radio waves formed by the data transmission equipment in space, a numerical model is constructed between the multi-level equipment and the transmission data. The numerical relationship can be expressed as follows:


(7) $$Y_{L}=\frac{\log\left(F_{f}\right)}{A}\times V$$

(8) $$A=\frac{N_{B}}{Z_{a\,b}}$$

In formula (7) and formula (8), *Y*_*L*_ represents the numerical transmission threshold. *F*_*f*_ represents the carrier frequency generated by simultaneous interpreting data at the time of data transmission, and *A* represents the attenuation factor of the data signal. *N*_*B*_ represents the path loss index of different transmission devices. The identification data have a certain period in the identification process. In the multi-level transmission process, from the beginning to the end of transmission, the actual transmission period is two times the acquisition period of data identification data (Puzzitiello et al., 2021; Wen and Liao, 2021). According to the value of transmission period, the data transmission rate generated in the return process of identification data is constructed. To control and identify the large information overhead caused by data transmission delay, an equalization threshold is set in the multi-level transmission process to coordinate the multi-level equipment to generate delay. The equalization threshold can be expressed as follows:


(9) $$V_{v}=\rho \times \frac{T}{n}\times Y_{L}$$

In formula (9), *V*_*v*_ represents the set equalization threshold, ρ represents the unit energy of transmitted data, *T* represents the return period of identification data, and the meaning of other parameters remains unchanged. Under the control of the set coordinated equilibrium value, the multi-level data transmission equipment is controlled as low effect, and the multi-level data transmission mechanism is set with this effect mode as the processing object (Daugherty et al., 2020; Trojian et al., 2022).

**FIGURE 4:**
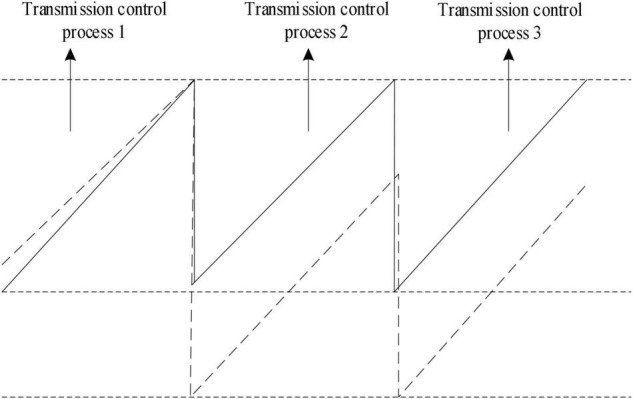
Multistage transmission control process.

Assuming that the multi-level transmission process is three levels, the numerical control process formed in the above transmission process is shown in Figure 4.

**FIGURE 5:**
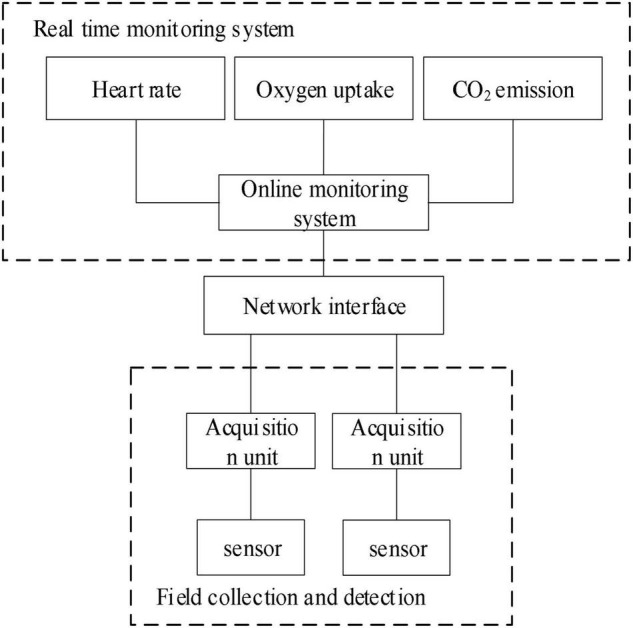
Information fusion processing of sports on mental health status recognition.

Corresponding to the transmission control process set in Figure 4, the sawtooth numerical model was called to fix the multi-level difference generated in the control process (Castle et al., 2021). In the set of multi-level transmission mechanism, corresponding to the protocol followed by the multi-level transmission process (Beynnon et al., 2020; Seow et al., 2020), the information fusion processing of sports on mental health status recognition is realized according to the index parameters such as maximum oxygen uptake VO2max and heart rate HR, as shown in Figure 5.

As shown in Figure 5, in the information fusion processing of sports on mental health status recognition, the psychological status of each individual is a multi-dimensional information system. Its basic characteristics are multivariable, multi-level, and strong disaster combination. There are complex non-linear interactions among various factors in the system. Due to the non-linear relationship of various factors, the evaluation of mental health is very suitable for deep learning. Considering the input and output relationships of recognition, it is in line with the characteristics of deep learning and finally realizes the information fusion processing of sport recognition of mental health state, so as to complete the recognition and analysis of sport recognition of mental health state based on deep learning.

## Experimental Analysis

**FIGURE 6:**
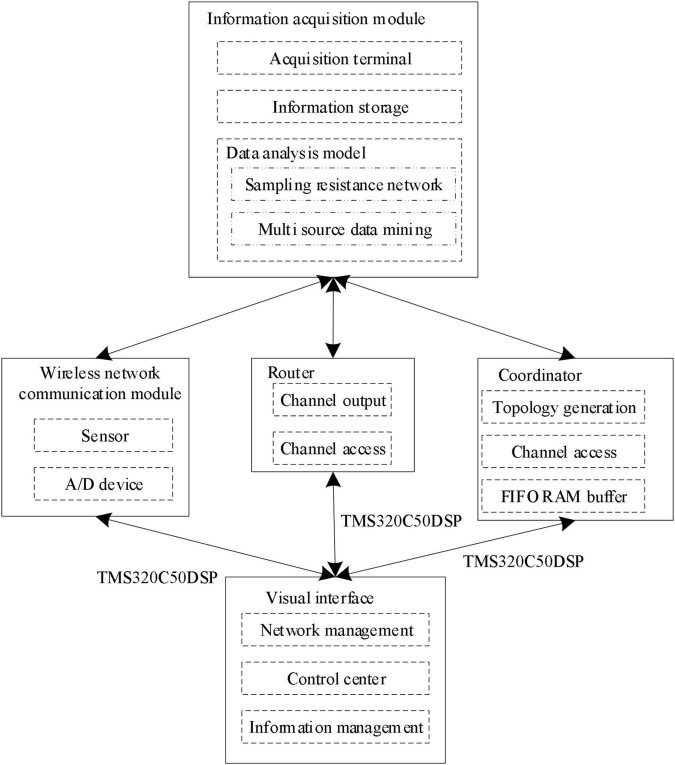
Technical framework of sport intensity information collection.

To verify the effect of sports based on deep learning on the recognition and analysis of mental health status, experiments were carried out. In the analysis of the recognition content of sports on mental health status, the wireless sensor network is used as the information acquisition module to build the data acquisition technical framework of sport intensity information. The results are shown in Figure 6.

In Figure 6, the sport intensity information is collected and stored through the acquisition terminal of the information acquisition module. After being processed by the data analysis model, it is uploaded to the PC interface through the wireless network, router, and coordinator to visually present the collected information. In the information acquisition module, multi-source data mining is applied to complete the information fusion of sport intensity. At the same time, the remote transmission control model is established with tms320c50dsp as the core chip to complete the integrated processing of sport intensity information. The FIFO RAM buffer of the overall architecture is triggered by sync or trigger to complete the control of instruction loading and information. The collected characteristic parameters of sport intensity information mainly include cardiac muscle strength, heart rate, cardiac blood supply, etc. Finally, the optimized sport intensity information is presented through the visual interface.

**FIGURE 7:**
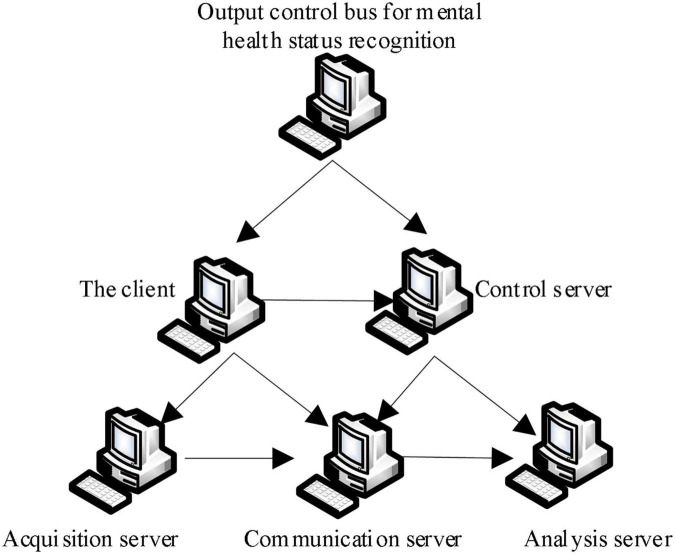
Simulation experiment environment diagram.

Combined with ZigBee networking, the Internet of things control module for mental health status recognition is constructed. The dm9000 of davicom company is used as the information processor, and max8660 is used as the output control bus for mental health status recognition. A PC with 8 g memory is selected for the experiment, and the simulation experiment is carried out with MATLAB 2012 software. The simulation environment is shown in Figure 7, and the parameters of the PC are shown in Table 1.

**TABLE 1:** Parameters of PC used in the experiment.

Serial number	Name	Parameter
1	Processor	Quad core 3.3 g
2	Graphics card	Independent graphics card 3 GB video memory
3	Memory	DDR3 4 GB
4	Hard disk	500 GB 7,200 RPM/SATA
5	USB	USB3.0
6	Power supply	Single power supply 220 V, 50 Hz

In the simulation experiment environment shown in Figure 7, the PC with the parameters as shown in Table 1 is used. The 2021 exercise health data of residents aged 30–40 in a community and the 2021 exercise health data of college students in a university as the experimental objects are collected and marked them as datasets A and B, respectively. This paper uses the model to identify the physical health patterns of residents and college students in the community after sports and analyzes the recognition effect of sports on mental health.

**FIGURE 8:**
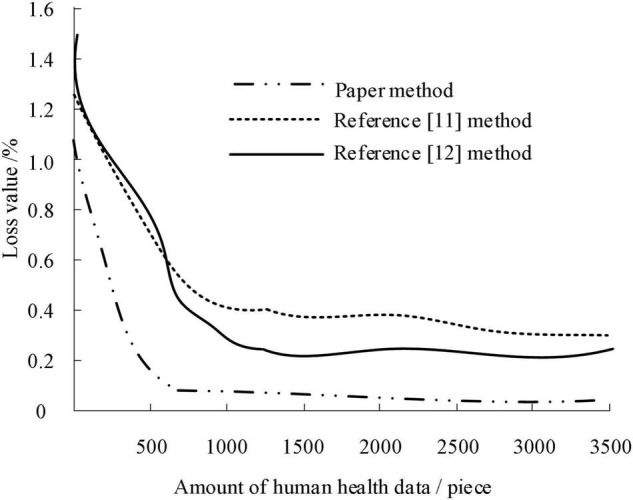
Reliability test results of three methods.

Taking the classification loss value when identifying the mental health model as the measurement index and dataset a as the experimental object, the change in classification loss value during identification under different mental health data samples is tested. To highlight the reliability of this method, the methods of Madsen et al. (2020) and Schweizer et al. (2020) are used to carry out the experiment. The results are shown in Figure 8.

According to the analysis of Figure 8, when analyzing the recognition of mental health state by sports, the loss value curve of the three methods decreases rapidly with the increase of the amount of mental health data before the amount of mental health data is 1,000, but when the amount of mental health data exceeds 1,000, the loss value curve of the three methods is not affected by the amount of mental health data, showing a relatively gentle trend. The maximum loss value of this method is lower than that of the literature method, and it begins to show a stable trend when the amount of data is about 600. The above results show that the loss value of this method in analyzing the effect of sports on mental health enhancement is the smallest, and the output result is reliable.

**FIGURE 9:**
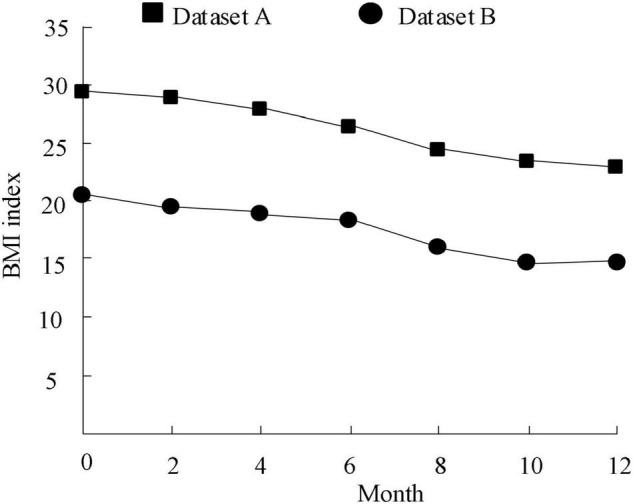
Changes of mental health index of community residents and college students.

Taking the mental health index BMI as the index and datasets A and B as the experimental objects, the model in this paper is used to output the changes in BMI values of community residents and college students when they exercise for 1 year. The results are shown in Figure 9.

According to the analysis of Figure 9, the mental health index of community residents and college students decreases with the increase of exercise months, and the mental health index of college students is lower than that of community residents. The initial BMI of college students and community residents exceeded 15, but after continuous sports, dataset A decreased to about 24 and dataset B decreased to about 15, which is the normal standard of human body. The above results show that continuous sports can effectively improve the BMI of human body and enhance physical fitness.

In the above experimental environment, the data in the multi-level transmission process are identified, and the data balance point of multi-level transmission control is used as the comparison index. When the transmission control method reaches the transmission balance, the data amount that can be controlled by the three multi-level transmission methods at one time is calculated, and the numerical relationship can be expressed as follows:


(10) $$P_{l}=W_{n}\times \left(A_{1}+B_{1}+C_{1}+D_{1}\right)$$

**FIGURE 10:**
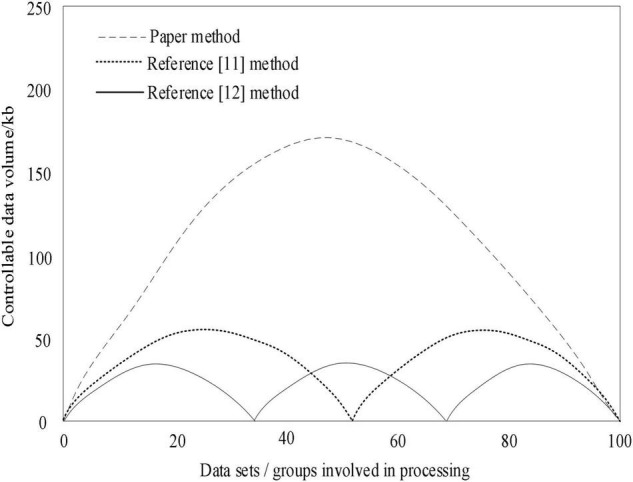
Comparison results of the amount of data that can be controlled by the three methods.

In formula (10), *A*_*1*_ represents the balance parameter, *B*_*1*_ represents the link parameter, *C*_*1*_ represents the balance parameter, *D*_*1*_ represents the priority parameter, and *W*_*n*_ represents the controllable amount of data. When the above numerical relationship is met, the amount of data generated will be balanced corresponding to the numerical value. Finally, the amount of data that can be controlled by the three multi-level transmission control methods is shown in Figure 10.

Under the constructed numerical relationship, when the three methods balance the processing of identification data, according to the experimental results shown in Figure 10, after processing ten sets of datasets at the same time, Schweizer et al. (2020) method controls three times in total, and the amount of data that can be processed at a single time is about 25 kb, which is less than the amount of data that can be actually controlled. The method in Madsen et al. (2020) controls two times in total. The amount of data that can be controlled in a single time is about 50 kb, and the actual amount of data that can be controlled is small. The designed method can process ten groups of datasets at a time, and the amount of data that can be processed at a single time is about 225 kb. Compared with the two existing methods, the designed method can control the most amount of data.

## Discussion

(1) Principle of positive emotion: the emotional state people experience in sports can directly affect their subjective wellbeing and interpersonal relationship, which are the important factors to promote people’s physical and mental health. The emotional state of sport instructors in sports can also directly affect the effect of mental health education. In the process of sport intervention in people’s mental health, we should maintain positive and full emotions and create a positive and safe environment.
(2) Experience success principle: strong successful experience is more likely to quickly and effectively improve people’s mental health. It is not only the basic way to improve people’s mental health, but also the way to achieve the modern “people-oriented” goal. In the process of identifying the mental health state of sports, we must always implement the principle of successful experience, so we must set sport goals reasonably. Therefore, appropriate sport goals should be established according to the physical abilities, interests, and hobbies of different groups, so as to ensure the experience of sport success, improve the healthy psychological state, and lay a foundation for the development of mental health.

(3) The healthy growth of people’s emotions in sports is driven by the principle of democratic autonomy, which originates from the general principle of sports. In people’s self-organized sports, the principle of democratic autonomy is also applicable. Under this self-organized activity, people can experience and gradually develop the awareness of social fairness of abiding by rules and norms, obtain social opportunities to communicate and communicate with peer groups, and then act on the final development of mental health.
(4) Individual counseling principle: because people’s age, physical quality, the basis of sport knowledge and skills, temperament and personality are different, there will be similarities and differences in psychological reflection and psychological performance in sports. In the arrangement of sports, we should consider the specific situation of each individual, teach students according to their aptitude, and pay attention to the development of individual mental health while cultivating sport ability. With reference to their emotional state, learning attitude, interpersonal relationship, and other performance make accurate judgment and carry out targeted counseling to guide people to be ideologically aware of their problems.

## Conclusion and Prospects

### Conclusion

Compared with previous research results, the main conclusions of this paper are as follows:

(1) The maximum loss value of the method in this paper is lower than that in the literature, and it begins to show a steady trend when the data volume is about 600. When analyzing the effect of sports on mental health enhancement, the loss value is the smallest, and the output results are more reliable.
(2) The mental health index of community residents and college students decreased with the increase of exercise months, and the mental health index of college students was lower than that of community residents. Continuous physical exercise can effectively improve the BMI and enhance physical fitness.
(3) After processing 10 sets of datasets at the same time, the designed method can process ten sets of data at a time, and the amount of data that can be processed at a time is about 225 kb, and the amount of data that can be controlled is the largest.

### Prospects

(1) Refine the research content: The next step is mainly to design the sport training path of mental health state from the theoretical level. For the verification or case study of the training path of mental health state of different training subjects, different ages, and different sports, it needs to be explored in the future.
(2) Expand the research object: In the future, the mental health status of other subjects will be extended, such as school-age students in vocational and technical high schools. In addition, the objects involved in providing education are school sport organizers. For groups such as personnel of education authorities and logistics support personnel who provide educational resources at the same time, it needs to be further included in future research.
(3) Increase empirical support: Continue to increase the empirical investigation data of the constructed training path and to further verify the operability of the path, some junior and senior high schools will be selected for detailed empirical investigation and in-depth case feasibility analysis in the future research.

## Data Availability Statement

The original contributions presented in this study are included in the article/supplementary material, further inquiries can be directed to the corresponding author/s.

## Author Contributions

Both authors listed have made a substantial, direct, and intellectual contribution to the work, and approved it for publication.

## Conflict of Interest

The authors declare that the research was conducted in the absence of any commercial or financial relationships that could be construed as a potential conflict of interest.

## Publisher’s Note

All claims expressed in this article are solely those of the authors and do not necessarily represent those of their affiliated organizations, or those of the publisher, the editors and the reviewers. Any product that may be evaluated in this article, or claim that may be made by its manufacturer, is not guaranteed or endorsed by the publisher.
